# Generative Protein Design: From Deep Learning Algorithms to Translational Applications

**DOI:** 10.3390/ijms27093917

**Published:** 2026-04-28

**Authors:** Shaotong Luo, Bo Zhou

**Affiliations:** 1 College of Aulin, Northeast Forestry University, Harbin 150040, China; 2023224789@nefu.edu.cn; 2 College of Life Science, Northeast Forestry University, Harbin 150040, China

**Keywords:** deep learning, protein design, SE(3)-equivariant, decoupled design, hybrid approaches, co-design

## Abstract

Deep learning has transformed protein design from a field long dominated by explicit energy-function optimization into one dominated by probabilistic generative modeling. In this review, we summarize the protein representation algorithmic basis for this transition, from sequence-centered encodings to geometric graph representations and, more recently, SE(3)-equivariant structural manifolds that directly respect three-dimensional symmetry. We classify current approaches into three methodological paradigms according to how sequence and structure are related during design: sequence–structure decoupled design, hybrid approaches, and sequence–structure co-design. For decoupled workflows, we discuss hallucination, backbone generation, and backbone-conditioned sequence design. For hybrid approaches, we examine integrated two-stage architectures and predictor-driven iterative co-refinement. For co-design, we review explicit joint generative formulations in which sequence and structure are treated as a coupled design state throughout generation. Additionally, we summarize evaluation principles for assessing the design results, such as physical validity, folding consistency, and design coverage, and then introduce some important applications in several fields. Taken together, these developments indicate that generative protein design is making progress from structure generation toward the programmable engineering of complex biological function.

## 1. Introduction

Protein design aims to discover amino acid sequences that not only adopt a desired three-dimensional fold, but also satisfy predefined functional requirements. This goal requires grasping a variety of complex rules, including thermodynamics, geometry and chemistry. 

For many years, the discipline addressed this challenge mainly through a physics-centered framework. Early de novo studies frequently employed simplified model systems to better elucidate the physical principles governing protein folding stability, including hydrophobic burial, secondary-structure propensity, and molecular packing regularity [1,2]. These principles were gradually refined into empirical scoring functions, thereby rendering computational protein design tractable. Within this framework, the design problem was formulated as an optimization task over residue identities and rotamer configurations, generally relying on a fixed or weakly flexible protein backbone [3,4]. Rosetta achieved a breakthrough in this era. By integrating fragment-based conformational sampling with Monte Carlo simulated annealing [5], Rosetta demonstrated that effective exploration of rugged energy landscapes was achievable, enabling the generation of novel topologies not previously observed in nature [6]. Nevertheless, it remained constrained by the inherent limitations of explicit energy functions and discrete optimization [7].

The situation changed with the rise of deep learning and the rapid expansion of protein sequence and structure data. Models such as AlphaFold2 and RoseTTAFold showed that neural networks could infer structural organization by extracting high-order evolutionary and geometric patterns directly from data, rather than relying exclusively on manually assembled physical terms [8,9].

The success of these predictive models suggested that protein design could be reformulated as a probabilistic learning problem, in which models learn the statistical regularities of natural proteins and use them to generate new candidates under specified constraints. Several recent reviews have already provided broad overviews of this rapidly evolving field [10,11,12,13]. Rather than revisiting that broader landscape, this work focuses on a more specific methodological question: how do current generative methods organize the relationship between sequence and structure during design? Viewed through this lens, existing approaches can be grouped into three main paradigms. In the first, sequence and structure are handled in a staged, factorized manner, with backbone generation and sequence design treated as sequential subproblems. In the second, models learn a fully joint generative distribution and treat sequence and structure as a coupled design state throughout the generation process. Between these two sits a third hybrid paradigm. These methods do not learn a fully joint generative distribution from the outset or keep sequence and structure completely separated. Instead, they coordinate the two during inference through shared intermediate representations, iterative refinement, or predictor-guided optimization.

This three-part framework provides a clearer view of the current methodological landscape than a simple binary division and serves as the main organizational axis of this review. Furthermore, we emphasize breakthrough developments over the past two years, highlighting the representative architectures that define the current frontier of the field.

## 2. Generative Protein Design Algorithms

### 2.1. Protein Representation and Geometric Deep Learning

Before comparing these design paradigms, it is useful to establish the representational and mathematical machinery on which they depend. Regardless of the downstream objective, AI-driven protein design requires translating a complex biological macromolecule into a form that a neural network can process without losing the structural regularities that matter most. Progress in the field has therefore been tightly linked to progress in representation learning. Over time, protein representations have evolved from one-dimensional sequence embeddings to three-dimensional topological graphs and then to explicitly geometric formalisms designed to respect the symmetries of Euclidean space. This progression reflects a growing recognition that proteins are sparse, structured objects whose geometry cannot be treated as generic data.

#### 2.1.1. 1D Sequence Semantics

Sequence is the most immediate representation because it is the linear carrier of evolutionary and biochemical information. In principle, the residue string specifies the energetic tendencies that shape the folding landscape and constrain which structures can be realized in a given environment. Protein language models adopt the self-supervised training strategy of natural language processing [14]. By masking residues in large evolutionary corpora and asking the model to recover them, these systems embed discrete amino acids into dense continuous representations. Their practical value comes from the fact that they capture nonlocal dependencies along the chain, including co-evolutionary signals between distant positions [15,16]. Even in the absence of explicit geometric coordinates, attention maps in deeper network layers still contain the structural information that can be used to infer residue–residue contact maps. This demonstrates that pure sequence models can distill structural principles directly from evolutionary data [17]. Therefore, it can be seen that the sequence semantics can play a role in the generative task, and can make up for the lack of physical constraints, especially in the absence of homologous templates [18,19,20].

#### 2.1.2. 3D Topological Graphs

**Figure 1:**
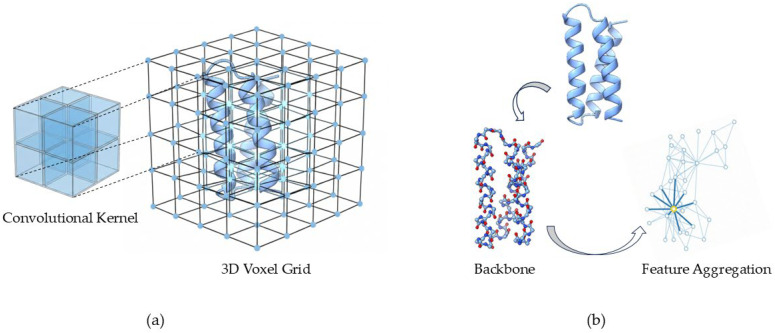
Structural representation strategies for 3D protein geometry. (**a**) 3D voxel grid representation and convolution. The 3D protein space is divided into a large number of regular grids, and the 3D-CNN is used to extract features. (**b**) Graph Neural Networks (GNNs). The protein is regarded as a topological graph, the node is the biological entity, and the edge can reflect the spatial relationship, which can be processed by the message passing mechanism.

When the design task requires the generation of 3D conformation, it is necessary to use a strategy to describe the Euclidean space. An early solution, inspired by computer vision, discretized local protein environments into regular volumetric grids and then applied 3D convolutional networks to those voxels [21,22]. However, this method has obvious shortcomings. First, the grid is too dense yet the physical structure of the protein is sparse, which leads to the problem of computational redundancy. Secondly, the model lacks rotation invariance and is too sensitive to input orientation, requiring data enhancement (Figure 1a). Consequently, graph-based representations are more common, which can abstract proteins into topological graphs, define atoms as nodes, and define spatial proximity as edges [23,24,25,26,27,28]. This modeling method can better meet the sparsity requirements of biomolecules and can quickly pass messages, which is conducive to the formation of long-range geometric interaction (Figure 1b).

#### 2.1.3. SE(3) Geometric Equivariance

Graphs solve the sparsity problem, but scalar graph features alone do not adequately encode directionality. For tasks that demand high spatial fidelity, such as chain packing and atomic-resolution generation, models must do more than remain invariant to rotations and translations. They must also be equivariant: when the input coordinate frame changes, the output should transform in a predictable covariant way rather than remaining unchanged or becoming inconsistent. This requirement has driven the development of SE(3)-equivariant architectures [29,30].

Two broad design philosophies are common. One is rooted in group representation theory. In that line of work, geometric features are expressed as irreducible representations of SO(3), decomposed into tensors of different angular degrees, and coupled through spherical harmonics, tensor products, and Clebsch–Gordan coefficients [31,32]. These constructions are mathematically elegant and highly expressive, especially for capturing fine angular detail, but they can be computationally demanding [33,34]. The other philosophy pursues a more pragmatic balance between rigor and efficiency. Rather than carrying out full tensor algebra at every stage, these models separate scalar and vector channels or use simplified geometric interactions that preserve essential equivariance while remaining more computationally scalable [24,35].

### 2.2. Sequence–Structure Decoupled Design

Early generative protein-design methods were largely built on a decoupled strategy, in which sequence and structure are handled as ordered subproblems rather than generated jointly. In practice, this framework developed along two main routes. One uses a structure predictor to guide sequence optimization directly, with the predictor serving as an external scoring function. The other follows a structure-first logic, in which a backbone is generated first and a compatible sequence is assigned afterward. Although later developments have made these pipelines more integrated, the core idea remains the same: sequence and structure are not generated as a single coupled state, but are instead linked through a staged design process.

#### 2.2.1. Predictor-Driven Hallucination

One prominent decoupled strategy is commonly referred to as hallucination. Its basic idea is to use a strong structure predictor as the scoring function that guides sequence search, effectively replacing classical biophysical energy terms [36,37,38,39,40,41]. In practice, optimization is performed directly in sequence space by gradient descent or by stochastic procedures such as Markov Chain Monte Carlo (MCMC), with the objective of maximizing predictor-derived confidence signals. This can yield sequences predicted to adopt novel folds. However, the method has an important weakness: optimization may exploit imperfections in the predictor itself, producing adversarial sequences that score well computationally but do not reliably fold under real experimental conditions.

#### 2.2.2. Backbone Coordinate Generation

**Figure 2:**
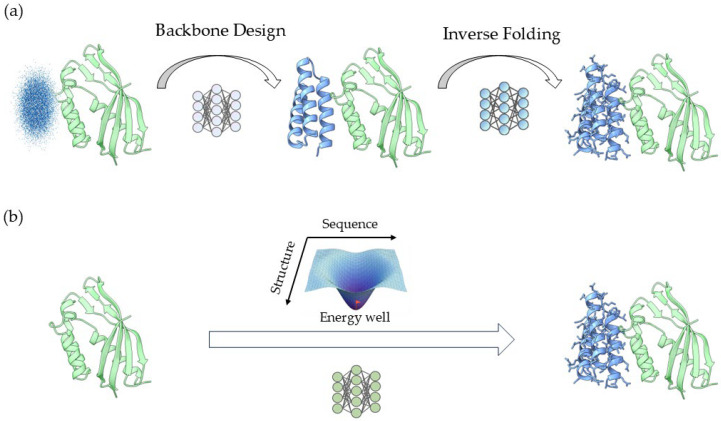
Mainstream paradigms in generative protein design. (**a**) Sequence–structure decoupled design. The task is decomposed into two ordered stages: backbone generation followed by backbone-conditioned sequence design (inverse folding). (**b**) Sequence–structure co-design. Sequence and structure are treated as a coupled design state and optimized within a shared or iterative framework so that mutually compatible solutions can emerge during inference. Hybrid approaches lie conceptually between these two strategies, but are not shown as a separate unified panel because they do not follow a common implementation principle.

To gain more explicit geometric control and reduce the risk of adversarial optimization, decoupled design also developed along a structure-first route. As summarized in Figure 2a, this strategy mathematically factorizes the high-dimensional joint generation problem into the product of a structural prior distribution $p\left(\mathrm{Structure}\right)$ and a conditional sequence distribution $p\left(\mathrm{Sequence}|\mathrm{Structure}\right)$ [42,43]. The first stage in this decomposition is backbone generation. The central challenge lies in parameterizing the continuous distribution of macromolecules in 3D Euclidean space while rigorously accounting for inherent roto-translational invariances and residue rigid-body constraints.

At the implementation level, three design choices are especially important. First, because protein backbone geometry lives on SE(3) rather than in a flat Euclidean space, the noising process is usually defined separately over translational and rotational degrees of freedom: positions are perturbed in Euclidean space, whereas orientations are diffused on SO(3) using geometry-aware stochastic processes [43,44,45]. This separation is necessary because rotational variables cannot be treated as ordinary vectors without violating the underlying geometry. Second, this noising scheme is closely related to coordinate parameterization. Instead of operating directly on all-atom coordinates, many backbone generators represent each residue as a local rigid frame constructed from the three non-collinear backbone atoms N, Cα, and C. This representation preserves local backbone geometry, reduces unnecessary degrees of freedom, and remains naturally compatible with SE(3)-equivariant learning. Third, the training objective is not only to recover a clean structure from a noisy input, but also to maintain global frame consistency during generation. For this reason, some influential implementations replace the frame-aligned loss commonly used in structure prediction, such as FAPE, with unaligned mean squared error (MSE), which is more suitable when generation does not assume a unique global reference frame.

A particularly influential design pattern in diffusion-based backbone generation is to initialize the denoising network from a pre-trained structure predictor that already contains strong geometric inductive biases and spatial reasoning capacity, and then fine-tune that model as a conditional generative module [43].

Beyond conventional stochastic diffusion, flow matching has emerged as an alternative formulation for structural generation. Instead of learning a reverse-time denoising process, it learns a deterministic ordinary differential equation (ODE) vector field that transports probability mass along smooth trajectories. This deterministic transport can improve numerical stability in sampling and appears particularly advantageous for scaling generation to very large proteins and multicomponent assemblies [46].

#### 2.2.3. Backbone-Conditional Sequence Design

After a plausible backbone has been obtained, the next task is to assign a compatible sequence—in other words, to solve inverse folding. This mechanism aims to accurately model the conditional probability $p\left(\mathrm{Sequence}|\mathrm{Structure}\right)$ [47]. Early neural inverse-folding methods framed this as a structure-constrained assignment problem on protein graphs. This shift allowed the field to move from hand-crafted energy functions to residue preferences learned directly from 3D geometry [23,25]. Later models improved structural encoding using geometry-aware message passing. These advancements led to highly effective inverse-folding architectures that estimate sequence likelihood directly from backbone coordinates, performing well in both monomeric and multichain design [26].

A major methodological improvement in this area is the shift away from rigid residue-generation orders. Instead of decoding strictly from the N-terminus to the C-terminus, many recent models use order-agnostic autoregression or masked reconstruction. These flexible decoding schemes are valuable because they capture long-range epistatic interactions more effectively. They also simplify practical design tasks, including multichain sequence design, partial sequence inpainting, and symmetry-constrained assignment [26,27,48,49].

Another important development has been the use of predicted structures to augment training data. Because experimental structures are scarce, high-confidence predicted structures can enlarge the effective training set and expose inverse folding models to a broader range of geometries. This augmentation has been especially helpful for improving zero-shot generalization to novel or de novo topologies [28].

Parallel to direct sequence probability prediction, an alternative technical route involves learning energy-based models (EBMs) over the sequence space. Rather than sampling residues directly from a decoder, these methods define an energy landscape over candidate sequences, with the landscape implicitly capturing both pairwise and higher-order interactions [50]. The decoupling of scoring and generation allows the vast sequence space to be explored through heuristic search algorithms or Markov chain Monte Carlo (MCMC) sampling. This formulation is especially attractive when the design target places unusual emphasis on fine-grained thermodynamic control or binding affinity [51].

### 2.3. Hybrid Approaches

Hybrid approaches occupy the methodological middle ground between staged decoupled design and fully joint co-design. Instead of keeping sequence and structure completely isolated or modeling them as a unified state from the start, these methods introduce partial coupling through shared internal representations or iterative inference.

#### 2.3.1. Integrated Two-Stage Design

Classic decoupled pipelines treat backbone generation and sequence assignment as separate modules. This modularity is convenient, but it introduces a distributional mismatch. The backbones produced by a generative model may lie outside the training distribution of the inverse folding model that follows, making sequence assignment less reliable.

To make up for these deficiencies, recent studies have introduced integrated two-stage design frameworks [42,52]. In these systems, backbone generation and sequence assignment remain distinct stages, but the boundary between them is softened by shared internal representations of spatial and sequence information. Methodologically, the key change is that sequence design no longer starts from a completed backbone alone, but from an intermediate structural state that already carries sequence-relevant information. This reduces interface mismatch while preserving the controllability of scaffold-first design. Recent models illustrate this shift through distinct architectural choices. While Chroma practically follows a backbone-first route, it integrates backbone generation with a dedicated design network that conditions sequence and side-chain generation directly on the sampled backbones, thereby tightening the connection between the two stages [38]. ODesign similarly preserves an explicit two-stage strategy, yet strengthens coordination through shared multimodal representations and conditional interaction modeling across its generative modules [44]. These systems therefore remain operationally staged, but are no longer fully decoupled in the classical sense.

#### 2.3.2. Predictor-Driven Iterative Co-Refinement

A second hybrid strategy also avoids learning a full joint generation. Instead, it treats a strong structure predictor as an implicit likelihood model and performs design by searching the induced landscape [8,53,54,55,56]. The predictor acts as an oracle for structural plausibility and constraint satisfaction, while the design algorithm updates the sequence in response to that oracle. Here, the coupling is introduced not through shared staged representations, but through repeated feedback during inference.

One route performs gradient-based optimization by backpropagating through the predictor to update sequence representations directly. Methods in this family differ mainly in how they relax the discreteness of amino acids. Relaxed Sequence Optimization (RSO), for example, keeps the sequence entirely in a continuous distributional form during optimization. The model therefore moves smoothly across a relaxed landscape until it converges on a favorable target backbone, after which an inverse-folding model is used to recover a physically discrete sequence for that optimized structure [57]. In contrast, BindCraft follows a different strategy. Instead of separating backbone optimization from sequence realization, it uses a staged optimization, which begins in continuous logit space, then gradually anneals toward one-hot sequence assignments [58]. With straight-through estimators and a final stage of explicit mutational sampling, BindCraft can extract discrete sequences directly from AlphaFold gradients. In both cases, complex multi-objective functions such as structural confidence (pLDDT), geometric consistency, and interface contact scores are rendered differentiable.

Another methodology avoids backpropagation altogether and instead performs iterative closed-loop design using only forward passes. In each iteration, the pipeline alternates between structure prediction and sequence design. The structure predictor utilizes its inherent “hallucination” capabilities to generate or refine a structure, which is then passed to an inverse folding model to redesign a highly compatible sequence. Recent frameworks like HalluDesign and Protein Hunter exemplify this approach. By alternating forward passes of structure refinement and inverse folding, they simultaneously enhance foldability and designability without relying on gradient descent [59,60]. While this block-coordinate style is easy to implement and often effective in practice, its fundamental limitation is that sequence–structure compatibility relies on repeated external predictor calls rather than being natively encoded within a joint generative prior.

### 2.4. Sequence–Structure Co-Design

In real proteins, sequence and structure are intrinsically coupled at every scale, as even minor sequence perturbations can reshape conformational preferences, while backbone geometry simultaneously restricts which residue patterns remain viable. Therefore, co-design aims to model sequence and structure jointly from the outset, as summarized in Figure 2b.

The explicit joint generative models aim to learn the joint distribution $p\left(\mathrm{Structure},\;\mathrm{Sequence}\right)$ so that covariation between sequence and geometry is represented natively rather than imposed only during downstream refinement. The central challenge is that the co-design model must accommodate discrete amino-acid identities, continuous geometry, and side chains with variable topology while preserving stereochemical realism and SE(3) equivariance. Different architectures implement this idea in different spaces.

In explicit all-atom approaches, the model operates directly in coordinate space, using atomic positions and residue rigid frames as the joint state to update sequence-related or structure-related variables along the shared generative trajectory [61,62,63,64]. To remain independent of the chosen coordinate frame, these models typically maintain SE(3) equivariance throughout. Their main technical obstacle is the variable dimensionality of amino-acid side chains. Because residues do not all possess the same number and arrangement of atoms, variable-topology chemistry must be embedded into a common computational representation if synchronous generation is to remain tractable. The advantage of this path is direct access to fine atomic interactions, including packing geometry and hydrogen-bond organization. The primary strength of this approach lies in its structural fidelity. By remaining entirely within atomic space, local packing, side-chain placement, and interface chemistry can be optimized concurrently with backbone geometry. However, this high resolution comes at the cost of computational complexity. Explicitly coupling sequence identity with all-atom coordinates substantially complicates training and sampling, particularly for targets involving ligands, modified residues, or multicomponent assemblies.

Semi-latent approaches keep backbone geometry explicit but compress residue identity and side-chain state into a fixed-dimensional continuous latent variable, establishing a unified generation process over the joint space [65,66]. By translating discrete sequence-related information into a continuous representation, this method allows noise injection and reverse updates to be performed stably in Euclidean space, thereby offering superior training stability and computational efficiency compared to explicit all-atom paths. These models offer a practical compromise between physical detail and computational ease. By keeping the backbone explicitly defined in 3D space while compressing sequence information into a continuous latent variable, they significantly improve numerical stability. The premise is that this latent space must retain enough chemical information to accurately reconstruct actual amino acids and side chains during decoding.

Fully latent approaches push the abstraction further. The entire sequence–structure state is encoded into a high-dimensional latent manifold, joint generation is learned in that latent space, and pretrained decoders are later used to reconstruct sequence and coordinates [67,68]. When the latent code is inherited from large pretrained structural models [16], it can also import substantial prior knowledge about sequence–structure coupling. Moving the generation process into a fully continuous latent space makes these models highly scalable, as it avoids the mathematical difficulty of mixing discrete sequences with continuous geometry. The unavoidable cost, however, is a loss of transparency and control. Because the representation is so abstract, it becomes nearly impossible to tell whether the model genuinely learned the physical relationship between sequence and structure, or simply offloaded that burden to the final decoder.

A more rigorous compromise between discrete sequence and continuous geometry is offered by multimodal flow-matching schemes. In these systems, discrete dynamics govern sequence evolution while continuous SE(3)-equivariant transport drives structural updates. By synchronizing these distinct streams through shared neural components, the framework successfully preserves the native dynamics of both modalities [69].

### 2.5. Evaluation Metrics

Evaluating generative protein design aims to determine whether generated candidates are physically valid, computationally consistent, and sufficiently diverse. Furthermore, different design paradigms demand tailored validation strategies. Decoupled pipelines must independently assess the structural prior and the conditional sequence model. Co-design architectures must verify the mutual consistency of the jointly generated state. Meanwhile, hybrid frameworks must ensure that predictor-guided coupling has not produced adversarial artifacts. Importantly, these in silico metrics do not correlate equally with experimental success. While some primarily serve to filter out clearly nonphysical outputs, others actively enrich for candidates more likely to fold or function, yet none can ultimately replace experimental validation.

#### 2.5.1. Physical Validity

Validity is the first screening layer. Its purpose is to determine whether generated outputs lie inside the manifold of physically possible folded proteins. For models that produce backbones or residue frames, this assessment focuses on geometric realizability, including chain continuity, the absence of severe steric self-intersections, and reasonable Ramachandran statistics. Global shape descriptors can also help identify clearly pathological topologies. For all-atom outputs, the criteria become stricter and include side-chain rotamer legality, bond length, angle deviations, and interatomic clash scores. Validity checks function as primary filters to discard biophysically impossible conformations, rather than direct proofs of “designability”

#### 2.5.2. Folding Consistency

Consistency provides the core computational evidence that a design actually satisfies the assumptions of the method that produced it. In inverse-folding studies, native sequence recovery (NSR) remains a widely used metric for quantifying residue-level agreement between designed and natural sequences [26,28]. However, NSR mostly measures how well a model recovers one plausible sequence under a conditional distribution, and its value depends strongly on local backbone quality [70]. High local recovery does not necessarily correspond to global stability, so it should not be interpreted as direct proof of global stability or experimental success [71].

A stronger closed-loop criterion is self-consistency, often treated as an in silico foldability test. In a typical protocol, the designed sequence is submitted to an independent structure predictor, and the resulting structure is compared against the intended target using measures such as scRMSD or scTM-score together with confidence indicators like pLDDT [26]. These metrics usually correlate more strongly with experimental tractability than sequence recovery alone, because they test whether the designed sequence returns to the intended structural basin. Even so, they remain enrichment criteria rather than direct surrogates for expression, stability, affinity, or function.

For co-design models, the notion of consistency extends naturally to cross-consistency. Here the sequence and structure generated together are re-evaluated as a pair: the sequence is refolded and the predicted structure is aligned back to the model’s own generated structure. This tests whether the output truly forms a coupled solution rather than an accidental combination of individually plausible components.

For hybrid approaches, especially predictor-guided iterative refinement frameworks, predictor confidence alone is an insufficient safeguard. Such pipelines are especially vulnerable to adversarial solutions that satisfy one oracle numerically while remaining physically suspect. Robust consistency evaluation therefore needs to incorporate sequence-prior plausibility and, ideally, orthogonal predictors during validation so that model-specific bias is reduced [19].

Accordingly, the relationship between in silico consistency metrics and experimental success should be understood as probabilistic rather than deterministic: better computational scores generally improve hit rates, but they do not guarantee experimental success.

#### 2.5.3. Design Coverage

Once validity and consistency have been established, evaluation shifts toward how broadly and how creatively a model explores the design space. Coverage is usually discussed in terms of diversity and novelty. Diversity reflects the range of solutions that can be produced under a fixed set of constraints and can be quantified by sequence clustering, pairwise identity, or structural fold-space coverage. Sampling hyperparameters are important here because they govern the practical trade-off between confidence and variety. Novelty, in turn, measures the distance between generated designs and known database entries, helping to distinguish genuine generalization from simple nearest-neighbor memorization.

## 3. Applications of Protein Design

### 3.1. Synthetic Biological Tools

Generative protein design has created many molecular tools and biosensors, which can play an important role in the development of synthetic circuit engineering, biological analysis and other fields.

For highly dynamic targets, the generative model can stabilize the recognition of a series of conformational ensembles to obtain binders that can bind to intrinsically disordered proteins (IDPs). These binders can be used to analyze dynamic targets [72]. In intracellular engineering, miniaturized binder modules have also been designed to occupy transient interfaces in DNA mismatch repair complexes. Acting as modular regulators, these binders improve prime editing efficiency and illustrate the value of de novo proteins as genetic engineering modules [73].

In the process of generative design, dynamic cell sensors and programmable switches can also be created. By computational design, the interface electrostatic repulsion is eliminated or the buried histidine network is constructed so that pH-sensitive binders can be created, which can be dissociated in the acidic environment, and the binding event can be controlled reversibly [74]. The use of advanced algorithms can accurately predict the optimal insertion site so that the receptor domain can be integrated into the effector protein, and the allosteric switch with a strong response ability can be obtained [75]. De novo design can not only produce single-chain allostery, but can also obtain modular protein oligomers, which can be assembled strictly in the presence of specific small-molecule drugs. The assembly of multiple domains under the induction of ligands can accurately control complex biological processes, including reversible condensate formation and spatial localization [76].

In the study of lipid bilayers, the design of synthetic molecules should have not only good hydrophobicity, but also strong signal transduction ability. The newly designed anion channels can respond to the electric field, and can play an important role in neuronal suppression [77]. Similarly, transmembrane fluorescence-activating proteins convert ligand binding into high-signal-to-noise optical readouts, thereby enabling distinct membrane-embedded biosensors [78].

### 3.2. Therapeutic Applications

Beyond tool development, generative protein design has also been extended to therapeutic intervention.

**Figure 3:**
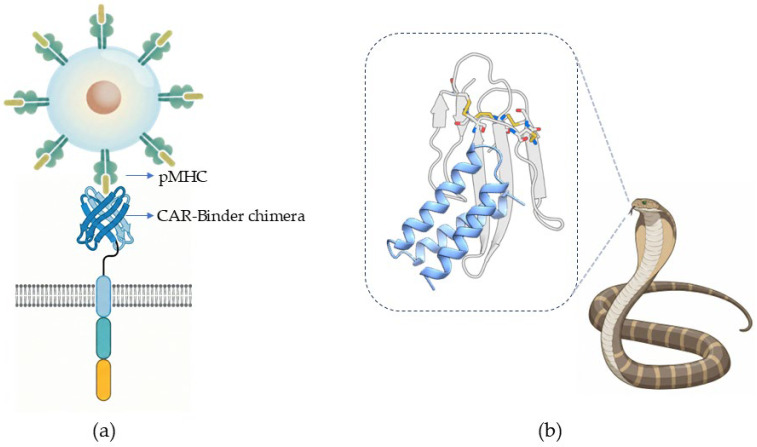
Therapeutic applications of de novo protein design. (**a**) Targeted immunotherapy using a CAR-binder chimera engineered to recognize peptide–major histocompatibility complex (pMHC) on the cell surface with high specificity. (**b**) Venom neutralization mediated by high-affinity de novo binders that inhibit lethal snake venom toxins.

In cancer immunotherapy, a central challenge lies in designing binders capable of discriminating between a specific disease-associated peptide–major histocompatibility complex (pMHC) and a vast repertoire of closely related complexes presented on the same HLA background. Because the underlying MHC scaffold is highly conserved, achieving exquisite specificity necessitates preferential recognition of the outward-facing residues of the presented peptide, rather than relying on extensive contacts with the MHC itself. To address this, recent computational strategies have engineered “peptide-centric” binding interfaces and enforced T-cell receptor (TCR)-like docking geometries. These approaches maximize peptide-focused recognition while minimizing off-target MHC cross-reactivity [79,80]. When integrated into chimeric antigen receptors (CARs) or other T-cell-engaging formats, these de novo interfaces mediate peptide-selective T-cell activation and robust cytotoxicity against target cancer cells, effectively translating precise molecular recognition into potent therapeutic function [81], as illustrated in Figure 3a.

Generative design has also enabled therapeutic control over immune signaling and receptor pharmacology. Soluble Notch agonists use programmed assembly geometries to induce non-natural receptor clustering, promoting T-cell development while reducing dependence on solid-phase presentation [82]. In cytokine therapy, engineered ultra-fast dissociation kinetics provide temporal control over signaling windows and help mitigate toxicities associated with sustained immune activation [83].

Related principles have also been extended to metabolic and membrane receptors, in which designed proteins modulate signaling by stabilizing specific functional states. Designed agonists can bias insulin receptor signaling by stabilizing specific allosteric states [84]. For G protein-coupled receptors (GPCRs), designed exoframe modulators target peripheral transmembrane interfaces and offer a state-selective approach for pharmacologically challenging receptors [85]. Similarly, de novo peptide modulators can target pathological states of endogenous sodium channels and restore inactivation gating, offering a potential therapeutic strategy for arrhythmias and epilepsy-associated electrophysiological dysfunction [86]. Designed proteins can also achieve the purpose of neutralization, which can maintain stability in a variety of complex biological fluids and neutralize highly variable snake venom toxins (Figure 3b) [87].

### 3.3. Enzyme Design and Catalysis

**Figure 4:**
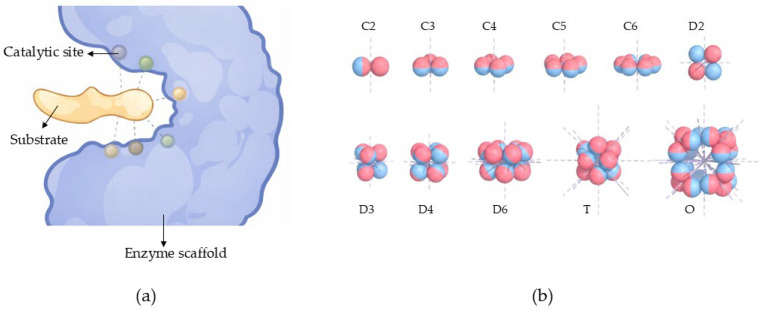
Design principles for catalytic proteins and protein materials. (**a**) Schematic illustration of enzyme design, where a substrate is positioned within an enzyme scaffold and surrounded by catalytic residues arranged with the high geometric precision required to drive the intended reaction. (**b**) Modular assembly of protein materials. By precisely programming interfacial geometries, basic subunits can be directed to form diverse symmetrical architectures, including cyclic (C2–C6), dihedral (D2–D6), tetrahedral (T), and octahedral (O) complexes.

Enzyme design represents a further escalation in difficulty because the objective is no longer merely binding or structural compatibility, but control over chemical transformation. In this context, foldability is necessary but far from sufficient. Catalytic success depends on precise active-site geometry, stabilization of transition states and intermediates, control over protonation networks, and favorable local electrostatics. As illustrated in Figure 4a, the central design problem is not simply to create a folded scaffold, but to position catalytic groups around the substrate with the spatial precision required for the target reaction.

Model reactions such as Kemp elimination have therefore played an outsized role as benchmarks [88]. They offer a chemically clear setting in which one can test whether a designed scaffold can place catalytic groups with the required accuracy. Computational strategies that tightly constrain transition-state geometry and electro-static arrangement have produced folded proteins with meaningful catalytic activity, sometimes approaching the lower edge of natural-enzyme performance in carefully defined cases. Harder targets, including serine hydrolases and metallohydrolases, demand additional control over catalytic triads, metal coordination, solvent structure, and multistep reaction pathways [89]. These studies show that modern design models can encode sophisticated chemical constraints, but they also make clear that catalysis places more severe demands on the design system than binding alone.

The incorporation of artificial cofactors extends this framework toward new-to-nature chemistry. By embedding synthetic cofactors such as porphyrins into designed backbones, researchers can bypass the chemical limits of natural side chains and enable efficient, stereoselective transformations of non-natural substrates [90]. In the cell environment, the combination of high-throughput directed evolution and computational design can not only solve the problem of substrate toxicity, but can also overcome the problem of cofactor assembly and cytoplasmic folding, and can achieve efficient artificial olefin metathesis [91].

### 3.4. Protein Materials

At a higher level of organization, generative protein design has opened new opportunities in protein materials, where higher-order function depends primarily on the precise design of assembly interfaces rather than on the complexity of individual monomers.

Compared with single-chain design, this area places greater emphasis on interface rigidity, geometric programmability, and the reusable combination of modular building blocks. One representative strategy is bond-centric design, which reduces complex three-dimensional assembly to the geometric programming of inter-component connection angles. Using a restricted library of structural modules, this framework enables access to a broad topological space spanning two-dimensional arrays, polyhedral nanocages, and three-dimensional lattices [92]. As summarized in Figure 4b, by controlling symmetry relationships and inter-subunit arrangements, design rules at the local interface level can be translated into well-defined higher-order architectures. In order to break through the constraints of point group symmetry, more advanced strategies introduce pseudo-symmetry and conformational symmetry breaking, generating bifaceted nanomaterials with built-in spatial heterogeneity [93].

Such topological foundations are naturally complementary to molecular recognition modules; by arranging binding domains with high positional precision and repeatability, designed protein materials support applications such as immunogen display, multi-target delivery systems, and nanoreactors.

## 4. Outlook

Generative protein design has progressed rapidly to a stage that de novo proteins can be engineered for binding, signaling, catalysis, and ordered assembly. Yet the central challenge has shifted. It is no longer enough to generate something that appears foldable in silico. What now matters is whether designed proteins can sustain robust function across the fluctuating, heterogeneous conditions of real biological environments.

One major limitation is that present-day workflows still depend heavily on structure predictors as practical scoring oracles for folding consistency. This strategy has obvious utility, but it also leaves design pipelines exposed to adversarial artifacts and model-specific biases. Future progress will therefore require complementary evaluation schemes that probe thermodynamic stability and biochemical affinity more directly. At the same time, most current methods remain anchored to static structural snapshots, whereas natural protein function often emerges from conformational fluctuation, state transitions, and environment-dependent interactions. A decisive next step will be to move from static-structure design toward the generative modeling of dynamic conformational ensembles, which is essential for allosteric proteins, molecular switches, and other adaptive systems.

Finally, the future design space is almost certainly larger than the present one. Incorporating non-canonical amino acids, nucleic acids, cofactors, and small-molecule ligands directly into generative models would expand both chemistry and function. As computational frameworks continue to absorb richer physical, chemical, and biological priors, generative protein design is likely to advance from simple protein-oriented construction toward the broader engineering of sophisticated biomolecules.

## Data Availability

No new data were created or analyzed in this study.
